# Protective Effect of Highly Polymeric A-Type Proanthocyanidins from Seed Shells of Japanese Horse Chestnut (*Aesculus turbinata* BLUME) against Light-Induced Oxidative Damage in Rat Retina

**DOI:** 10.3390/nu10050593

**Published:** 2018-05-10

**Authors:** Tomoe Ishihara, Sachiko Kaidzu, Hideto Kimura, Yasurou Koyama, Yotaro Matsuoka, Akihiro Ohira

**Affiliations:** 1 Department of Ophthalmology, School of Medicine, Shimane University, 89-1 Enya-cho, Izumo, Shimane 693-8501, Japan; t-ishihara@kozuchi-net.jp (T.I.); kecha@med.shimane-u.ac.jp (S.K.); ykoyama@med.shimane-u.ac.jp (Y.K.); ymatsu@med.shimane-u.ac.jp (Y.M.); 2 Department of Research and Development, Kotobuki Seika Co., Ltd., 2028 Hatagasaki, Yonago, Tottori 683-0845, Japan; h-kimura@kozuchi-net.jp

**Keywords:** retina protection, Japanese horse chestnut, *Aesculus turbinata* BLUME, polyphenolic compound, antioxidant, proanthocyanidin

## Abstract

Retinal tissue is exposed to oxidative stress caused by visible light. Light-damaged rat used in age-related macular degeneration (AMD) studies clarified that antioxidants decrease retinal light damage. Albino rats were exposed to 5000 Lux light for 12 h with oral administration of the polyphenolic compounds fraction (PF) from the seed shells of Japanese horse chestnut (30 mg/kg, 100 mg/kg, and 300 mg/kg body weight: BW). To evaluate the protective effects against light damage, electroretinograms (ERGs), the outer nuclear layer (ONL) thickness, the antioxidant activity of plasma, oxidized retinal lipids, and the detection of apoptosis were examined. To reveal their active compounds, PF were separated into an A-type proanthocyanidin (PAF) and a flavonol *O*-glycosides fraction. The protective effects of these fractions against light damage were compared by measuring the thickness of the ERGs and ONL. Compared with the negative control, the PF group (100 mg/kg and 300 mg/kg BW) significantly suppressed the decrease of the ERG amplitudes and ONL thickness. PF (300 mg/kg BW) induced the elevation of in vivo antioxidant activity, and the suppression of retinal lipid oxidation. PF administration also suppressed apoptotic cell death. The protective effects against light damage were attributable to the antioxidant activity of PAF. The light-induced damage of retinas was protected by oral administration of PF and PAF. Taken together, these compounds are potentially useful for the prevention of the disease caused by light exposure. Highlights: The protective effects of retinal damage by light exposure were evaluated using polyphenolic compounds from the seed shells of Japanese horse chestnut (*Aesculus turbinata* BLUME) as an antioxidant. Decreases in the electroretinographic amplitude and outer nuclear layer thickness were suppressed by the polyphenolic compounds of the seed shells. Polyphenolic compounds from the seed shells of Japanese horse chestnut inhibited the oxidation of retinal lipids. Highly polymeric A-type proanthocyanidin from the seed shells protected the rat retina from light exposure damage by inhibiting oxidative stress and apoptotic mechanisms.

## 1. Introduction

The seeds of the Japanese horse chestnut (*Aesculus turbinata* BLUME), which has been used as emergency food since antiquity, is an ingredient in rice cakes and rice balls. We reported previously that the seed shells of the Japanese horse chestnut, which is regarded as a waste byproduct, contain a large amount of polyphenolic compounds with different degrees of polymerization, i.e., highly polymeric A-type proanthocyanidins (PAFs) [1] and flavonol *O*-glycosides [2], which have antioxidant activity [1,2].

Age-related macular degeneration (AMD) results from aging and long-term light exposure. AMD is a progressive blinding disease with no cure at present. About 11 million individuals suffer from AMD in the United States alone, with a global prevalence of 170 million. AMD is thereby the leading cause of worldwide blindness [3].

Injection of anti-vascular endothelial growth factor drugs is one AMD treatment, but long-term treatment causes retinal atrophy [4]. There is no specific medical treatment for AMD. To delay the progression of AMD and visual loss, experimental and clinical studies have suggested that high doses of antioxidant vitamins and zinc supplements are potential strategies [5,6]. Therefore, preventive medicine is required, especially in countries with an aging population.

Retinal tissue has the highest oxidative consumption and consists of a unique fatty acid component [7]. Oxidized retinal phospholipids increase with aging [8]. These also substantially increase in the retinas of patients with AMD more than in normal retinal tissue [8]. The Age-Related Eye Disease Study (AREDS) revealed that the intake of antioxidant suppresses the progression of AMD [5]. These evidences indicate that oxidative stress might cause AMD [5]. Intense visible light also causes increases in lipid peroxidation and retinal damage [9]. A rat model of light damage used in AMD studies has clarified that antioxidants decrease retinal light damage [10].

In this study, we examined the protective effect of polyphenolic compounds from seed shells of Japanese horse chestnut using a rat model of retinal light damage.

## 2. Materials and Methods

### 2.1. Materials

The seeds of the Japanese horse chestnut (*A. turbinata* BLUME) were purchased from Takaki Co. (Kurashiki, Japan). Paper filter No. 2 was purchased from Advantec (Tokyo, Japan). A DIAION HP-20 and Chromatorex ODS 1024T for column chromatography were supplied by Mitsubishi Chemical (Tokyo, Japan) and Fuji Silysia (Kasugai, Japan). The Sephadex LH-20 for column chromatography was obtained from GE Healthcare (Tokyo, Japan). *N*-tert-butyl-α-phenylnitrone (PBN) was obtained from Sigma (St. Louis, MO, USA). Saline and 0.5% tropicamide and 0.5% phenylephrine hydrochloride eye drops were obtained from Otsuka Pharmacy (Tokyo, Japan) and Santen Pharmaceutical (Osaka, Japan). White fluorescent light (TL5 HE) was purchased from Philips Lighting (Tokyo, Japan). Reduced sugar syrup (PO-30) was obtained from Towa Chemical (Tokyo, Japan). An ApopTag Peroxidase In Situ Apoptosis Detection Kit, 3′-3′-diaminobenzidine, and a Lipid Hydroperoxide (LPO) Assay Kit were obtained from Chemicon (Temecula, CA, USA), Dako (Carpinteria, CA, USA), and Cayman Chemical (Ann Arbor, MI, USA), respectively. Hematoxylin and eosin (H&E) were obtained from Sakura Finetek (Tokyo, Japan). Methanol, acetone, and other chemicals of analytic grade were supplied by Wako Pure Chemical Industries Ltd. (Osaka, Japan).

### 2.2. Extraction and Purification of Polyphenolic Compounds

Extraction and purification of polyphenolic compounds from the seed shells of the Japanese horse chestnut were performed according to our previously reported procedures [1]. The seed shells (15.1 g, dry weight) were refluxed by boiling for 2 h in 1 L of distilled water. The mixture was filtered through Advantec No. 2 filters to obtain the extracts of the polyphenolic compounds. The solvent was removed by rotary evaporation in vacuo and resulted in a 2.72 g yield of dry material. To remove sugars, proteins, and lipids, the extracted material was subjected to column chromatography on the DIAION HP-20 (470 × 60 mm inner diameter (i.d.)) and eluted with 500 mL of methanol after washing with 1 L of distilled water. The resulting methanol extracts were evaporated to dryness and dissolved in 5% methanol. For further purification, the aliquots were subjected to column chromatography on the Chromatorex ODS 1024T (330 × 40 mm i.d.). After the column was washed with 500 mL of 5% methanol, the polyphenolic compounds were eluted with 500 mL of 50% methanol and evaporated to dryness. This dry material (polyphenolic compounds fraction; PF) was redissolved in a small aliquot of ethanol and subjected to column chromatography on the Sephadex LH-20 (150 × 30 mm i.d.). The column was eluted serially with 500 mL each of ethanol, methanol, and 70% acetone to derive a flavonol *O*-glycosides fraction (FF), Pronathocyanidins Fraction 1 (PAF1), and Proanthocyanidin Fraction 2 (PAF2), respectively.

### 2.3. Animals

All of the procedures were carried out according to the Association for Research in Vision and Ophthalmology (ARVO) Statement for the Use of Animals in Ophthalmic and Vision Research. The Institutional Animal Care and Use Committees of Shimane University reviewed and approved all protocols. Five-week-old male Sprague-Dawley (SD) rats were obtained from Charles River Laboratories Japan, Inc. (Kanagawa, Japan) and maintained in a colony room at 22 °C for one week before the experiments. All of the rats were kept in a 12-h (7 a.m. to 7 p.m.) light/dark cycle. The light condition of the room was 80 lux.

### 2.4. Evaluation of Protective Effect of Polyphenolic Compounds Fraction (PF) against Light-Induced Retinal Damage in Rats

PF was dissolved in 50% (*w*/*w*) PO-30 aqueous solution. To administrate quantitatively, the PF solution was administered after the rats fasted for 3 h, using a stomach tube once daily orally into the stomach of the rats for seven days (four days before and three days after light exposure for 12 h at 5000 lux). The doses of PF were 30 mg/kg, 100 mg/kg, and 300 mg/kg of body weight. The group of PF (300 mg/kg) administration without light exposure was used as the sample control and the group that was administered solvent with light exposure was used as the negative control. PBN as the positive control was used to compare the protective effect of PF. PBN dissolved in saline was intraperitoneally injected into the rats 30 min before light exposure, as described previously [11,12] at doses of 10 mg/kg and 50 mg/kg of body weight. In addition, PBN cannot be administered orally. For this reason, our group examined the retinal protective effect of intraperitoneally-administered PF and PBN against light damage. As the result, we clarified that PF and PBN both had a protective effect (data not shown).

Thirty minutes after the fourth PF dose, the rats were exposed to intense light, as previously reported with slight modification [13]. All of the exposure to light began at 6 pm. The pupils were dilated with 0.5% tropicamide and 0.5% phenylephrine hydrochloride eye drops 15 min before light exposure. The rats were exposed to 5000 lux of diffuse, cool, white fluorescent light (TL5 HE) while housed for 12 h in clear plastic cages with wire tops. During exposure, the rats had free access to food and agar as a substitute for water. After exposure, the animals were returned to the dim cyclic light conditions.

Flash electroretinograms (ERGs) were recorded seven days after light exposure using an ERG recording system (LS-W, Mayo Corporation, Aichi, Japan). All of the animals were dark-adapted overnight before the measurement. Anesthesia was induced by intramuscular injection of a mixture of ketamine (100 mg/kg) and xylazine (20 mg/kg). The pupils were dilated with 0.5% tropicamide and 0.5% phenylephrine hydrochloride eye drops. Light-emitting diodes (LED) electrodes were placed on both eyes. An identical reference electrode was placed in the mouth; a ground electrode was placed on the tail. A single flashlight (10,000 cd/mm^2^, 5 ms) from the LED was used as the light stimulus. The a-wave amplitude was measured as the difference in voltage between the baseline value before the flash and the peak of the a-wave; the b-wave was measured as the difference in voltage between the peaks of the a-wave and b-wave. The a-wave and b-wave amplitudes obtained from the right and left eyes were averaged in each animal.

It is clear that photoreceptor cell in the outer nuclear layer (ONL) is damaged by intense light, and ONL thinning is progressed. Therefore, ONL thickness was measured in the retinal sections as described previously with slight modification [14] after ERG measurement. After euthanasia by an overdose of anesthesia and then by cervical dislocation, both eyes were enucleated and fixed in 4% paraformaldehyde containing 20% isopropanol, 2% trichloroacetic acid, and 2% zinc chloride for 24 h at room temperature. After alcohol dehydration, the eyes were embedded in paraffin, and 4-µm-thick sagittal sections containing the whole retina including the optic nerve head (ONH) were cut. The sections were stained with H&E. For each section, digitized images of the entire retina were captured with a digital imaging system (Eclipse E800, Nikon, Tokyo, Japan) at ×100 magnification. To cover the entire retina, 10 images were obtained from each section. The ONL thicknesses were measured at 0.5 mm, 1.0 mm, 1.5 mm, 2.0 mm, 2.5 mm, and 3.0 mm superior and inferior to the ONH and at the periphery 100 µm from the superior and inferior edges of the retina (peri) using ImageJ 1.32 software (National Eye Institute, Bethesda, MD, USA). The thickness values obtained from the right and left eyes were averaged for each animal. To compare each experimental group, ONL thickness was evaluated by area under the curve (AUC), i.e., area surrounded by the curve of the graph (Figure 3B) and x-axis.

### 2.5. Evaluation of Antioxidant Capacity of Rat Plasma

To analyze levels of oxidation stress, antioxidant capacity and potential antioxidant capacity of whole body, diacron-reactive oxygen metabolites (d-ROM) and biological antioxidant potential (BAP) tests of plasma were performed, and the BAP/d-ROM ratio was evaluated. Light exposure, dosage, and dosage methods of PF were the same as mentioned previously. Thereafter, 2% heparin-added whole blood was withdrawn from the tail vein at the day 1, day 4 (before light exposure), day 5 (after the light exposure), and day 12. The blood samples were centrifuged at 5000× *g* for 5 min to obtain the supernatants as the plasma samples. The analysis was performed using a free-radical analyzer system (FREE Carpe Diem, Wismerll Company Ltd., Tokyo, Japan) [15].

### 2.6. Evaluation of Lipid Oxidation of the Retina

The light exposure, dosage, and dosage methods of PF and PBN were the same as mentioned previously. Twenty-four hours after light exposure, the rats were sacrificed under anesthesia and perfused with cold phosphate-buffered saline. The retinas were collected and preserved at −80 °C until the LPO assay. The retinas were homogenized in 0.1 M of acetate buffer (pH 5.5), and subjected to LPO analysis using an LPO Assay Kit [16].

### 2.7. Apoptosis Detection by TUNEL Staining

PF was dissolved in 50% (*w*/*w*) PO-30 aqueous solution. To administrate quantitatively, the PF solution was administered after the rats fasted for 3 h, using a stomach tube once daily orally into the stomach of the rats for five days. The doses of PF and PBN were 300 mg/kg and 50 mg/kg of body weight, respectively. The PBN dissolved in saline was intraperitoneally injected into the rats 30 min before light exposure (5000 lux, 12 h). Forty-eight hours after light exposure, the rats were sacrificed under anesthesia and the eyes were enucleated and fixed as described previously. TdT-mediated dUTP nick end labeling (TUNEL) staining was performed with a kit (ApopTag Peroxidase In Situ Apoptosis Detection Kit) on 4-µm-thick section at 1 mm superior to the ONH of all of the eyes. 3′-3′-diaminobenzidine was used as a chromogen.

### 2.8. Evaluation of the Protective Effect of Flavonol O-Glycosides Fraction (FF) and Proanthocyanidin Fraction (PAF) Against Light-Induced Retinal Damage in Rats

The experimental conditions were the same as described previously. FF and PAF were dissolved in 50% (*w*/*w*) PO-30 aqueous solution. The doses of FF and PAF were both 300 mg/kg of body weight.

### 2.9. Statistical Analysis

The data are expressed as the mean ± standard error of the mean in each group (*n* = 4–6). The statistical significance was evaluated by one-way analysis of variance (ANOVA) with Mini StatMate software (ATMS Co., Tokyo, Japan) followed by the post-hoc Tukey’s test and Dunnett’s test. *p* < 0.05 was considered statistically significant. The post-hoc Tukey’s test was used to analyze statistical difference between groups. Dunnett’s test was used to analyze statistical difference to negative control.

## 3. Results

### 3.1. Extraction and Purification of Polyphenolic Compounds

**Figure 1:**
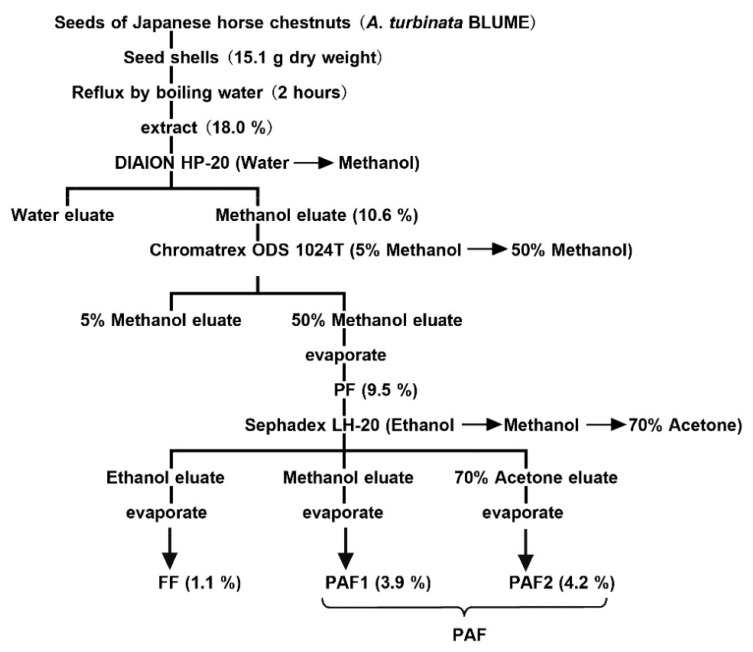
Extraction and purification procedure for a polyphenolic compounds fraction (PF), flavonol *O*-glycosides fraction (FF), and proanthocyanidin (PAF) from the seed shells of Japanese horse chestnut.

PF (1.43 g DW) was separated by DIAION HP-20 and Chromatorex ODS 1024T column chromatography from the water extracts of seed shells (2.72 g DW). The PF was separated further by column chromatography with Sephadex LH-20 into three fractions: FF eluting with ethanol, PAF1 with methanol, and PAF2 with 70% acetone (Figure 1). In this result, dry weight of FF, PAF1, and PAF2 were 0.17 g, 0.59 g, and 0.63 g, respectively. Recently, we reported that the polyphenolic compounds from the seed shells of Japanese horse chestnuts contained flavonol *O*-glycosides [2] and A-type proanthocyanidins. FF contained only flavonol *O*-glycosides. On the other hand, although the degrees of polymerization differed, PAF1 and PAF2 were contained A-type proanthocyanidins [1]. For this reason, PAF1 and PAF2 were mixed (PAF) and used for testing.

### 3.2. Evaluation of the Protective Effect of PF against Light-Induced Retinal Damage in Rats

**Figure 2:**
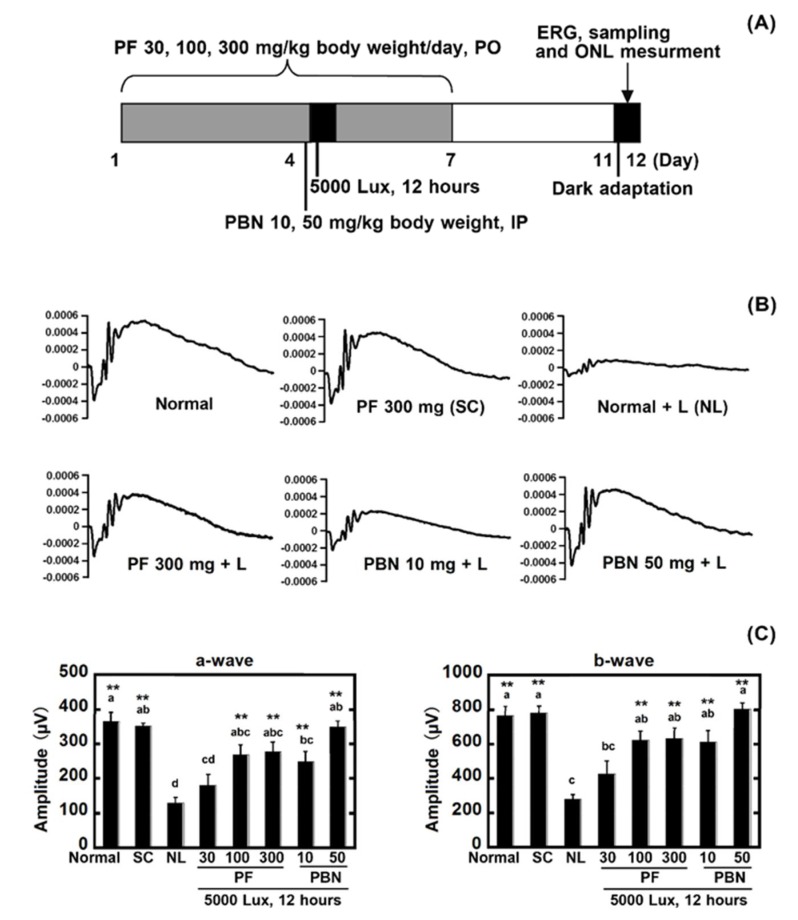
Protective effect of polyphenolic compounds fraction (PF) against light-induced retinal functional damage. (**A**) Experimental design. Rats were given oral PF (30 mg/kg, 100 mg/kg, and 300 mg/kg body weight/day) once daily for seven days (four days before and three days after light exposure). Intraperitoneal injections of *N*-tert-butyl-α-phenylnitrone (PBN) (10 mg/kg and 50 mg/kg body weight) were administered 30 min before light exposure. On the fourth day, the rats were exposed to intense light (5000 lux) for 12 h, after which they were returned to the dim cyclic light conditions; (**B**) Flash electroretinograms (ERGs) were recorded seven days after light exposure; (**C**) ERG amplitudes. The left graph shows the a-wave, and the right graph shows the b-wave. Normal indicates normal control, no light exposure, untreated; SC, sample control, no light exposure, administration of PF 300 mg/kg of body weight; NL, negative control, light exposure, administration of solvent; PF 30, light exposure, administration of PF 30 mg/kg of body weight; PF 100, light exposure, administration of PF 100 mg/kg of body weight; PF 300, light exposure, administration of PF 300 mg/kg of body weight; PBN 10, light exposure, injection of PBN 10 mg/kg of body weight; PBN 50, light exposure, injection of PBN 50 mg/kg of body weight; and PO indicates per os. Data represent the mean ± SEM (*n* = 6). These data show the results of analysis by Tukey’s method and Dunnett’s method. Alphabets (a, b and c) indicate values that differ significantly between each group (*p* < 0.05) tested by the Tukey’s method. Asterisks indicate values that differ significantly to negative control tested by the Dunnett’s method (* *p* < 0.05, ** *p* < 0.01).

To evaluate the protective effect of PF against light-induced retinal damage, the rats received oral PF for seven days (Figure 2A). After light exposure, a significant decrease of the ERG a-wave and b-wave amplitudes was observed in the group that did not receive PF (negative control; NL) compared with the normal control (Figure 2B,C). However, the retinal damage was suppressed significantly (*p* < 0.05) in the rats treated with the PF doses of 100 mg/kg and 300 mg/kg. The doses of 10 mg/kg and 50 mg/kg of intraperitoneally injected PBN (positive control) suppressed the retinal damage compared with the negative control. There was no significant difference between the normal control rats and those in the 300 mg/kg group without light exposure (Figure 2B,C).

**Figure 3:**
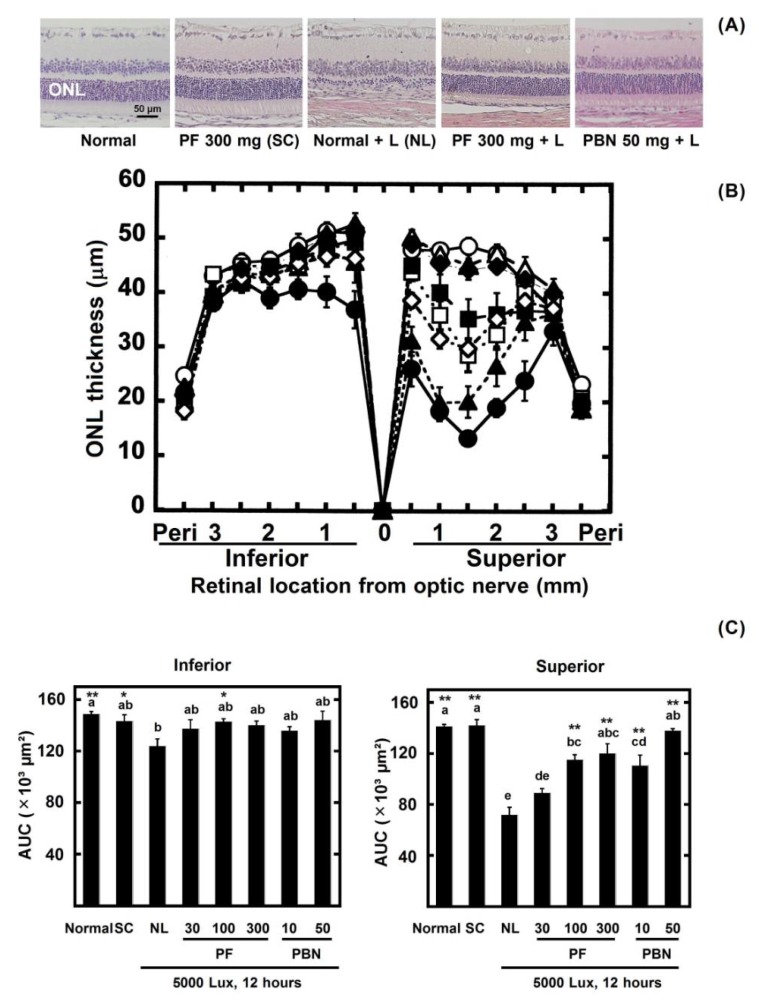
Protective effect of PF against light-induced retinal histologic damage. After ERG measurement, the ONL thickness was measured in the retinal sections. (**A**) Representative Hematoxylin and eosin (H&E) staining of the retinal sections were 1 mm superior to the outer nuclear layer (ONH); (**B**) The ONL thickness in each experimental group is shown. The data are expressed as the mean ± standard error of the mean (*n* = 6). Normal (○) indicates normal control, no light exposure, untreated; SC (△) indicates sample control, no light exposure, administration of PF 300 mg/kg of body weight; NL (●) indicates negative control, light exposure, administration of solvent; PF 30 (▲) indicates light exposure, administration of PF 30 mg/kg of body weight; PF 100 (□) indicates light exposure, administration of PF 100 mg/kg of body weight; PF 300 (■) indicates light exposure, administration of PF 300 mg/kg of body weight; PBN 10 (◇) indicates light exposure, injection of PBN 10 mg/kg of body weight; PBN 50 (◆) indicates light exposure, injection of PBN 50 mg/kg of body weight; and peri indicates the periphery 100 µm from the superior and inferior edges of the retina; (**C**) Area under the curve (AUC) of Figure 3B. Left graph indicates inferior, right graph indicates superior. Data represent the mean ± SEM (*n* = 6). These data show the results of analysis by Tukey’s method and Dunnett’s method. Alphabets (a, b, c, d, and e) indicate values that differ significantly between each group (*p* < 0.05) tested by Tukey’s method. Asterisks indicate values that differ significantly to the negative control tested by Dunnett’s method (* *p* < 0.05, ** *p* < 0.01).

The ONL thickness decreased in the light-exposed retina, especially in the superior retina. However, the result of the ONL AUC shows that there was slight damage in the PF-treated rats. The AUC of the rats treated with the PF doses of 100 mg/kg and 300 mg/kg was larger than the 10 mg/kg PBN groups, but not the 50 mg/kg PBN groups (Figure 3). 

### 3.3. Evaluation of Oxidative Stress and Antioxidant Capacity of Rat Plasma

**Figure 4:**
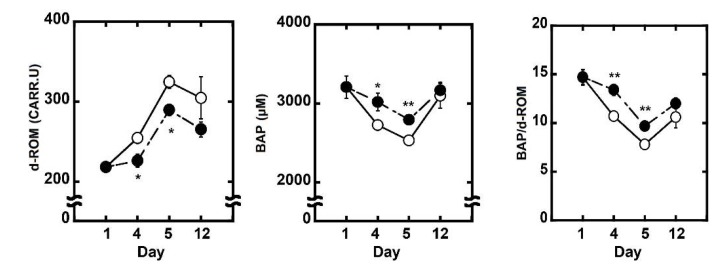
Evaluation of potential antioxidant capacity of rat plasma. Oxidative stress of plasma was evaluated by diacron-reactive oxygen metabolites (d-ROM) test. Antioxidant capacity was evaluated by the biological antioxidant potential (BAP) test. BAP/d-ROM showed potential antioxidant capacity. (**A**) The level of d-ROM; (**B**) The level of BAP; (**C**) The level of the BAP/d-ROM. NL (○) indicates negative control, light exposure, administration of solvent; and PF (●) indicates light exposure, administration of PF 300 mg/kg of body weight. Data represent the mean ± SEM (*n* = 6). An asterisk indicates a significant difference between negative control and PF (* *p* < 0.05, ** *p* < 0.01) followed by Dunnett’s test.

Four and five days after starting administration, 300 mg/kg PF lowered d-ROM levels of plasma and increased the level of BAP significantly. These data showed that PF raised the potential antioxidant capacity of whole body (Figure 4).

### 3.4. Evaluation of Oxidation in Retinal Lipids

**Figure 5:**
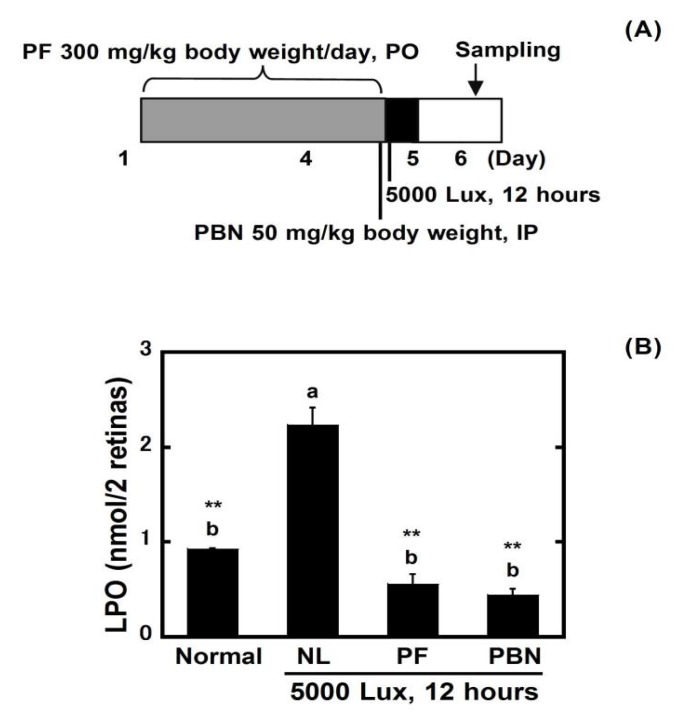
Inhibition of light-induced retinal lipid peroxidation. (**A**) Experimental design. The rats received oral PF (300 mg/kg of body weight/day) once daily for four days. PBN (50 mg/kg body weight) was administered 30 min before light exposure by intraperitoneal injection. On the fourth day, the rats were exposed to intense light (5000 lux) for 12 h, after which they were returned to the dim cyclic light conditions. The eyes were enucleated 24 h after light exposure. PO indicates per os; (**B**) The result of the lipid hydroperoxide (LPO) assay. Data represent the mean ± SEM (*n* = 4). These data show the results of analysis by Tukey’s method and Dunnett’s method. Alphabets (a and b) indicate values that differ significantly between each group (*p* < 0.05) tested by Tukey’s method. Asterisks indicate values that differ significantly to negative control tested by Dunnett’s method (* *p* < 0.05, ** *p* < 0.01).

A significant (*p* < 0.05) increase in the LPO levels occurred in the retina 24 h after exposure, which was suppressed by PF and PBN (Figure 5).

### 3.5. Apoptosis after Light Exposure

**Figure 6:**
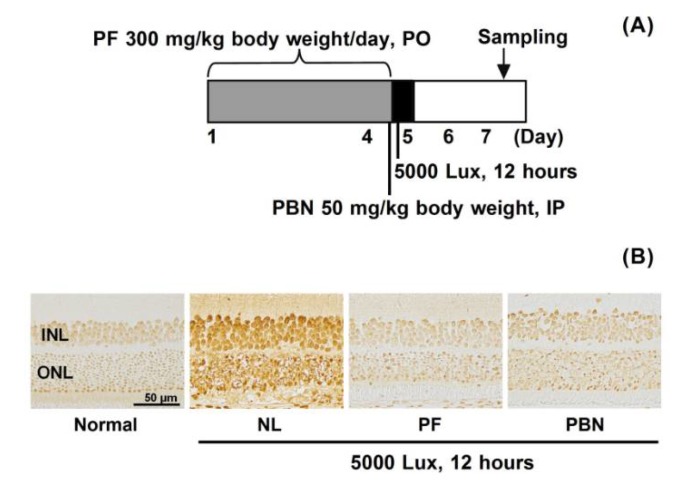
Inhibition of light-induced retinal cell apoptosis. (**A**) Experimental design (*n* = 4). The rats received oral PF (300 mg/kg of body weight/day) once daily for four days. PBN (50 mg/kg of body weight) was administered 30 min before light exposure by intraperitoneal (IP) injection. On the fourth day, the rats were exposed to intense light (5000 lux) for 12 h, after which they were returned to the dim cyclic light conditions. The eyes were enucleated 48 h after light exposure. PO indicates per os; (**B**) Representative TdT-mediated dUTP nick end labeling (TUNEL) staining images are shown.

In normal retinal tissue, there were a few apoptosis-positive cells; however, after light exposure, the number of apoptosis-positive cells increased in the inner nuclear layer and ONL. In the PF-treated and PBN-treated and light-exposed retina, a slight increase in the numbers of apoptosis-positive cells was seen, but there were fewer than in the untreated group (Figure 6).

### 3.6. Protective Effect of FF and PAF against Light-Induced Retinal Damage

**Figure 7:**
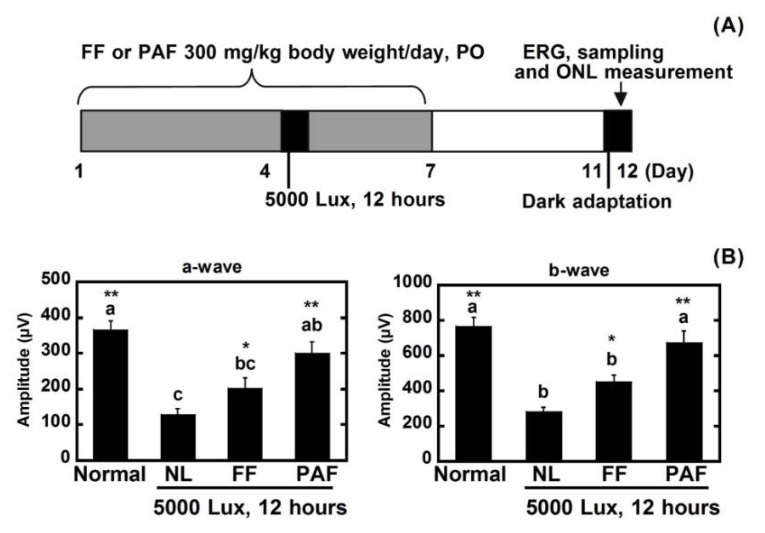
Evaluation of FF and PAF against light damage. (**A**) Experimental design. The rats received oral FF or PAF (300 mg/kg of body weight/day) once daily for seven days (four days before and three days after light exposure). Intraperitoneal injections of PBN (10 mg/kg and 50 mg/kg body weight) were administered 30 min before light exposure. On the fourth day, the rats were exposed to intense light (5000 lux) for 12 h. After light exposure, the animals were returned to the dim cyclic light conditions; (**B**) ERG amplitude. The left graph indicates the a-wave and the right graph indicates the b-wave. Normal indicates the normal control, no light exposure, untreated; NL indicates the negative control, light exposure, administration of solvent; FF indicates light exposure, administration of 300 mg/kg of body weight; PAF indicates light exposure, administration of 300 mg/kg of body weight; and PO indicates per os. Data represent the mean ± SEM (*n* = 6). These data show the results of analysis by Tukey’s method and Dunnett’s method. Alphabets (a, b, and c) indicate values that differ significantly between each group (*p* < 0.05) tested by Tukey’s method. Asterisks indicate values that differ significantly to the negative control tested by Dunnett’s method (* *p* < 0.05, ** *p* < 0.01).

**Figure 8:**
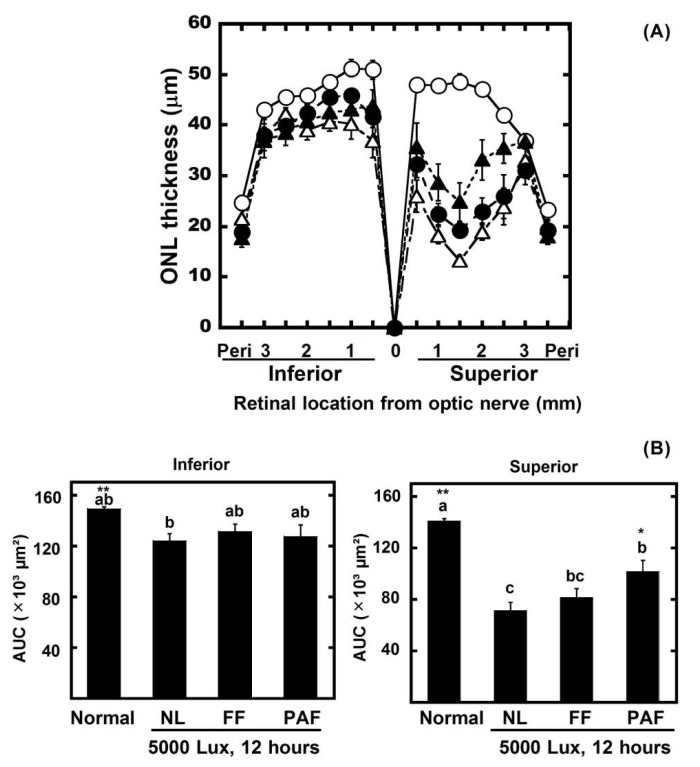
Protective effects of FF and PAF against light-induced retinal histologic damage. After ERG measurement, the ONL thickness was measured in the retinal sections. (**A**) The ONL thickness in each experimental group is shown. The data are expressed as the mean ± standard error of the mean (*n* = 6). Normal (○) indicates normal control, no light exposure, untreated; NL (△) indicates negative control, light exposure, administration of solvent; FF 300 (●) indicates light exposure, administration of FF 300 mg/kg of body weight; PAF 300 (▲) indicates light exposure, administration of PAF 300 mg/kg of body weight; and peri indicates the periphery 100 µm from the superior and inferior edges of the retina; (**B**) Area under the curve (AUC) of Figure 8A. The left graph indicates inferior, and the right graph indicates superior. Data represent the mean ± SEM (*n* = 6). These data show the results of analysis by Tukey’s method and Dunnett’s method. Alphabets (a, b, and c) indicate values that differ significantly between each group (*p* < 0.05) tested by Tukey’s method. Asterisks indicate values that differ significantly to the negative control tested by Dunnett’s method (* *p* < 0.05, ** *p* < 0.01).

To identify the compounds with a protective effect against light damage on the retina, 300 mg/kg of body weight of FF and PAF were compared with the negative control (NL) and normal control (Figure 7). FF contains flavonol *O*-glycosides [2]. However, although the degrees of polymerization differed, PAF1 and PAF2 are comprised of highly polymeric A-type proanthocyanidins [1]. For this reason, PAF1 and PAF2 were mixed (PAF) and used for testing. PAF suppressed the decreases in the ERGs and the ONL AUC on superior retinas. By Dunnett’s test, FF also significantly suppressed the damage of ERG compared with NL. The results indicated that PAF was more effective than FF for protecting against light damage (Figure 7 and Figure 8).

## 4. Discussion

We found that polyphenolic compounds from the seed shells of Japanese horse chestnut protect eyes from light damage. In this study, marked damage occurred in the ERGs and ONL as the result of strong light exposure. The ERG was measured to estimate the ocular function. The group exposed to 5000 lux light for 12 h clearly lost ERG function (60% loss versus normal) (Figure 2). The ONL thickness was measured to evaluate the retinal tissue. ONL thinning progressed as the result of light damage (30% loss) (Figure 3). However, the ERGs performed after PF treatment in rats showed a 30% loss, and 10% of the ONL thickness was lost. Therefore, the results suggested that PF protected eyes from light-induced damage. Moreover, to reveal the active compounds that protect against light damage in the retina, FF and PAF were separated from PF and used in animal experiments. PAF protected retinas against light-induced damage as the result of ERG and ONL. On the other hand, FF inhibited the decrease of ERG slightly compared with PAF. However, Dunnett’s test showed that both FF and PAF suppressed it (Figure 7 and Figure 8). On the other hand, we used PBN as a positive control in the retinal light damage model, but PBN is not approved for human use because it cannot be administered orally.

We reported that FF contained quercetin-3-*O*-[β-d-glucopyranosyl(1→3)]-[β-d-xylopyranosyl (1→2)]-β-d-glucopyranoside-3′-*O*-β-d-glucopyranoside, quercetin-3-*O*-[β-d-xylopyranosyl(1→2)]-β-d-glucopyranoside-3′-*O*-β-d-glucopyranoside, quercetin-3-*O*-[β-d-glucopyranosyl(1→3)]-[β-d-xylopyranosyl(1→2)]-β-d-glucopyranoside, quercetin-3-*O*-[β-d-xylopyranosyl(1→2)]-β-d-gluco pyranoside, quercetin-3-*O*-[β-d-xylopyranosyl(1→2)]-β-d-glucopyranoside-3′-*O*-[6-*O*-(nicotinoyl)-β-d-glucopyranoside],kaempferol-3-*O*-[β-d-glucopyranosyl(1→3)]-[β-d-xylopyranosyl(1→2)]-β-d-glucopyranoside, kaempferol-3-*O*-[β-d-xylopyranosyl(1→2)]-β-d-glucopyranoside, quercetin-3-*O*-[β-d-xylopyranosyl(1→2)]-β-d-glucopyranoside-3′-*O*-[6-*O*-(indolin-2-one-3-hydroxy-3-acetyl)-β-d-glucopyranoside [2]. Those eight flavonol *O*-glycosides have antioxidant activity [2]. Therefore, the flavonol *O*-glycosides contributed the retinal protective effect of FF. Our group previously showed that the structure of PAF was characterized through analyses combining high performance liquid chromatography (HPLC), matrix-assisted laser desorption ionization time-of-flight mass spectrometry (MALDI-TOF/MS), and liquid chromatography-electrospray ionization-mass spectrometry (LC-ESI/MS) [1]. PAF was highly polymeric A-type proanthocyanidins and contained 85% (*w*/*w*) in PF. Therefore, PAF was the main contributor that protected against light-induced damage.

Recently, the retinal protective effects against light damage of oral bilberry extract (containing about 39% anthocyanin) [17] and epigallocatechin gallate [18] have been reported. However, in those experiments, bilberry extract and epigallocatechin gallate were administered at doses of 750 mg/kg and 400 mg/kg BW, respectively. On the other hand, PF was effective using a lower dose than those experiments. It is reported that proanthocyanidins from the seed of sea backthorn protect against light damage in the retina [19]. In the study, rabbits were administered proanthocyanidins (50 mg/kg and 100 mg/kg BW) for three weeks. After light exposure, the ERG, ONL, malondialdehyde (MDA), catalase (CAT), glutathione peroxidase (GSH-Px) and pro-apoptotic protein (Bax) of retinas were measured. As the result, proanthocyanidins protected the retina by reducing oxidative stress and apoptosis. They used the crude extracts containing 38.9% of proanthocyanidin. Therefore, the active compounds for retinal protective effects were unclear. In our study, purified A-type proanthocyanidin was used, and then the active compound of protecting against light-induced damage was revealed. The absorption of proanthocyanidin depends on the degree of polymerization. Dimers and trimers were clearly absorbed through an intestinal epithelium cell monolayer [20,21]. While the absorption of these polymers was difficult, they were degraded by the colonic microflora into compounds of low molecular weight and absorbed [20]. These metabolites of proanthocyanidin were hydroxybenzoic acid, protocatechuic acid, hydroxyl phenylacetic acid, and so on [22]. Due to the antioxidant activities of these phenolic acids, retinas might be protected from oxidative stress by light exposure.

In this study, PAF treatment restored a-wave and b-wave amplitudes to control levels (Figure 7B). However, retinal morphology did not return to normal in this treatment group (Figure 8B). There was contradiction between results of the ERGs and the ONL. In the past studies, it was clarified that ONL thinning strongly occurred in the superior retina more than the inferior retina [23,24]. In contrast, the ERGs showed whole retinal function. Therefore, it was thought that the morphological data could disagree with the physiology data. However, it was certain that PAF significantly suppressed light damage both morphologically and histologically compared with negative control.

Retinal degeneration caused by genetic or external factors is produced by the oxidative damage of lipids, protein, and nucleic acids, resulting in apoptotic cell death. These phenomena have been observed after intense light exposure [25,26]. 

From the above results, it is revealed that PF, which has antioxidant activity, protects the retina from light damage. Therefore, PF was evaluated by LPO assay to clarify whether it suppressed lipid oxidation of the retina. Thirteen-hydroperoxy-9,11-octadecadienoic acid was used as the standard. In this research, LPO was 0.9 ± 0.0 nmol/retina in normal rats, and there were no changes in the LPO at 0 h and 12 h after light exposure (data not shown). At 24 h after light exposure, a significant (*p* < 0.05) increase of oxidized lipid occurred (2.2 ± 0.2 nmol/retina); however, oral administration of PF (0.6 ± 0.1 nmol/retina) suppressed the increase (Figure 5). An evaluation of plasma antioxidant capacity showed that the oral administration of PF raised the potential antioxidant capacity of the whole body (Figure 4). These results indicate that PF inhibits lipid peroxidation from active oxygen by increasing the antioxidant capacity of whole body, and therefore reduces the oxidative stress caused by light exposure and inhibits retinal light damage. 

It is clear that oxidation after light exposure is an early event preceding apoptosis [27]. To show whether apoptosis is involved in light damage and PF inhibits it, apoptosis was evaluated by TUNEL staining with the sample collected 48 h after light exposure. Apoptosis-positive cells increased in untreated retina by light exposure (Figure 6), but decreased with PF treatment, which indicated that PF suppresses apoptosis. A previous study reported that caspase-3 increased as the result of light damage via activating MAPK signaling, leading to apoptosis [28]. Quercetin, kaempferol, and grape seed proanthocyanidin inhibit apoptosis by reducing caspase activity [29,30,31]. In the current study, PF contained mainly highly polymeric A-type proanthocyanidins with antioxidant activity [1]. Considering them, PF might suppress apoptosis via the caspase cascade, which is a subject for future analysis.

It is reported that oxidized phospholipids increase in the retinas of AMD patients [8]. Oxidative stress has been suggested to be one of AMD causes. The results of LPO assay showed that PF suppressed lipid oxidation in retina. There is no specific medical treatment for AMD; hence, the intake of antioxidants is efficient for the prevention of AMD [5]. It is suggested that PF, which is derived from plant and is possible to be taken orally, is efficient material for the prevention of AMD.

## 5. Conclusions

In summary, the highly polymeric A-type proanthocyanidin of Japanese horse chestnut seed shells protect the retina from light exposure damage by inhibiting oxidative stress and apoptosis. Consequently, intake of supplementary Japanese horse chestnut seed shells appears to have a protective effect on the retina from diseases caused by oxidative stress such as AMD.
